# User-centered evaluation of Discord in midwifery education during the COVID-19 pandemic: Analysis of the adaptation of the tool to student needs

**DOI:** 10.18332/ejm/142638

**Published:** 2021-11-02

**Authors:** Lionel Di Marco

**Affiliations:** 1 Midwifery Department, Faculty of Medicine, Grenoble-Alpes University, Grenoble, France; 2 TIMC-IMAG Lab, UMR 5525, ThEMAS Team, CNRS, Public Health Department, Grenoble-Alpes University, Grenoble, France

**Keywords:** midwifery education, learning environment, confinement, social presence

## Abstract

**INTRODUCTION:**

In the context of the COVID-19 pandemic in 2020, and in order to overcome the lack of face-to-face contact between students and teachers, the midwifery department of Grenoble (France) decided to use the Discord tool in the training of midwifery students. In order to evaluate the relevance of using instant messaging software for the education of future midwives, the tool was evaluated by the students.

**METHODS:**

We conducted, in January 2021, a user-centered online study with all midwifery students in training for the classes of 2020–2021, using the French translation of Anstey and Watson’s Rubric for the Evaluation of eLearning Tools. This evaluation analyzed the different dimensions of Discord in the context of training: functionality, accessibility, technology, design, privacy and data protection, social presence, pedagogical presence, and cognitive presence.

**RESULTS:**

Discord had a good functionality for 75% of the students surveyed. They found Discord to be suitable for maintaining social links and creating serious games. But they did not find it useful for following courses or practical work. More than 80% of the midwifery students interviewed agreed that Discord can be adapted to different learning contexts.

**CONCLUSIONS:**

The department can continue to use Discord without reservation for the creation of serious games, as well as for maintaining links between students and teaching staff in the department. Discord has the characteristics of a social network, allowing students to connect with each other.

## INTRODUCTION

The COVID-19 pandemic has profoundly changed societies. Higher education has not been an exception to the changes imposed by more than a year of successive confinements.

In France, as of 17 March 2020, all university institutions had to modify their learning paradigms by having to find solutions to train all students at a distance^1^.

The Grenoble-Alpes University, and in particular the Faculty of Medicine, fortunately already had the technical means to implement such teaching. Indeed, since 2007, in the Department of Midwifery, part of the teaching has been carried out in a hybrid format, including flipped classrooms for about one-third of the teaching modules, all teaching units combined^2^.

In France, studies last 5 years, and from the beginning of the curriculum, students benefit from teaching focused on clinical reasoning, and the proposed educational activities are diverse and varied: lectures, question and answer sessions, learning clinical reasoning, problem-based approach, serious games, role-playing, high-fidelity simulation, etc.

Yet the existence of a system in place prior to the confinement did not prevent the need to compensate for the lack of face-to-face interaction with students. Thus, the pedagogical team proposed to use the Discord tool, well known in some digital communities^3^.

Discord is an application and a website that allows communities to communicate in various formats (text, video, audio) in a synchronous or asynchronous way. Historically used by online gaming communities, the tool has opened up to many other types of communities^4^.

It has been implemented in the training of midwifery students of all classes since the end of March 2020. We had therefore decided to conduct a user-centered evaluation of this tool in January 2021 in the context of the training of midwifery students in the Grenoble-Alpes University (France). The objective of this evaluation was to determine whether Discord met the standards of software dedicated to learning, and to evaluate the satisfaction of students with its use.

## METHODS

In order to determine whether Discord met these standards, we conducted a user-centered study of all midwifery students in training for the classes of 2020–2021. We decided to use Anstey and Watson’s Rubric for eLearning Tool Evaluation^5^, licensed under a Creative Commons Attribution-Noncommercial-Share-Alike 4.0 International License.

The declarative survey was conducted between January and February 2021, with 2 reminders in order to obtain a correct participation rate. All students in the department (n=151) were asked to complete the questionnaire, without any obligation to do so. No exclusion criteria were applied. No demographic data or information that could directly or indirectly identify the students were collected, in order to allow for the most candid responses possible from the students, without pressure from the training institute.

The rubric was created to allow instructors to analyze the relevance of a tool for learning. It offers the possibility of matching student needs with the offer of technologies for learning. This evaluation tool includes 8 dimensions broken down into 27 criteria.

Using the French translation of Schneider^6^, we were able to propose a questionnaire for students to give their views about the use of the tool in their learning. The following dimensions were analyzed:

- Functionality: to know if the tool meets expectations;
- Accessibility: to know if the tool can be effectively used in its context;
- Technical: to know if the technical specifications are effective;
- Mobile design: to know if all tools allow to use the software in good conditions; and
- Privacy, data protection and rights: to know if students are bothered by the tool in terms of personal data.

The last three dimensions were taken from the community of inquiry model^7^, these were:

- Social presence: to know if the tool facilitates learners’ ability to project their personal characteristics into the learning community;
- Teaching presence: to know if the tool supports trainers in designing, facilitating and directing educational processes; and
- Cognitive presence: to know if the tool facilitates the ability to construct meaning through communication.

Finally, we analyzed the uses of the tool. The questionnaire was administered digitally via the application LimeSurvey provided of the University of Grenoble-Alpes.

Participation in this study was voluntary. No direct or indirect identifying data were collected. The usercentered questionnaire used was only an opinion and satisfaction questionnaire on the use of software. The study’s information letter stated that students were free to participate or not in the study, and that it was not possible for the investigator to know who had or had not participated. No ethical considerations were therefore raised by the implementation of this study.

## RESULTS

The participation rate was 55.6%; 84 midwife students responded to the survey. All descriptive statistics were calculated on the number of respondents to the questionnaire (n=84). All questionnaires that were started were completed: there were no missing data in our study.

### Midwife students using Discord

Most students used a Discord server: 73% used the Midwifery Department’s server; 20% used another Discord server; 5% were non-users of Discord; and 2% did not know about Discord. Half of declared users (53.8%) had used more than one version of Discord (browser-based, mobile or tablet application, Windows, Linux or MacOS computer application) including 64% who did not find major differences between these versions.

They mostly used Discord for serious gaming, to stay in touch with their classmates, and to stay in touch with the department’s teaching staff.

### Functionality

**Figure 1:**
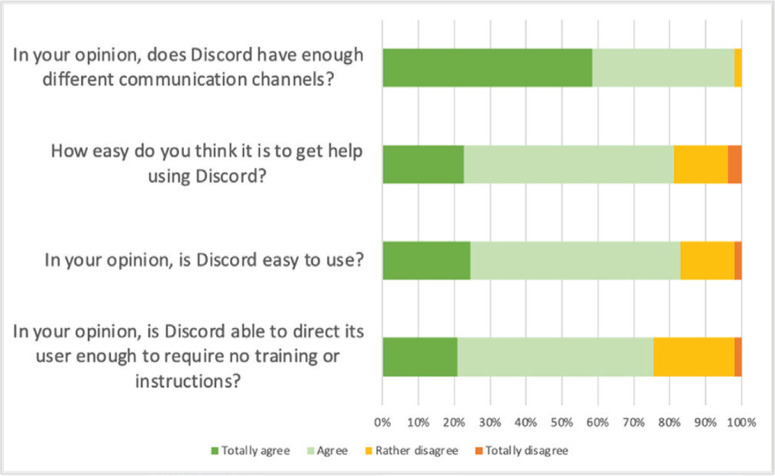
Perception of functionality of Discord for midwifery student users

Discord was a suitable tool whatever the size of the learner’s class for 88.7% of the respondents (47 students). Overall, Discord had good functionality for students: between 75% and 99% of respondents thought it had enough ways to communicate, was easy to use, and did not require special training (Figure 1).

### Accessibility and design

**Figure 2:**
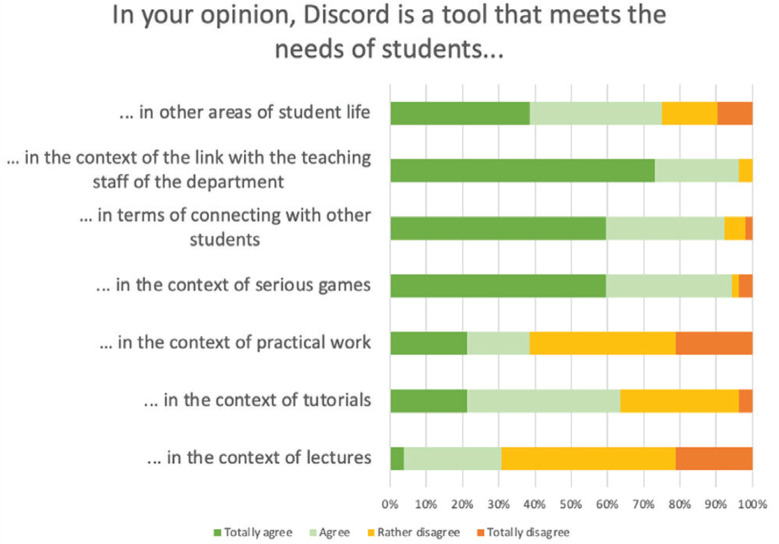
Usefulness of Discord during midwifery studies

Most of the participating students (96%) owned one or more devices that allowed them to use Discord. Two students (3.8%) did not own one, but had easy access to equipment that allowed them to use Discord. One student had difficulty accessing equipment to use Discord. Regarding usefulness, the view was that Discord was suitable for maintaining social ties and for making serious games. On the other hand, it was not regarded useful for following lectures or practical work. The opinions were more mixed concerning tutorials (Figure 2).

### Technology and data

**Figure 3:**
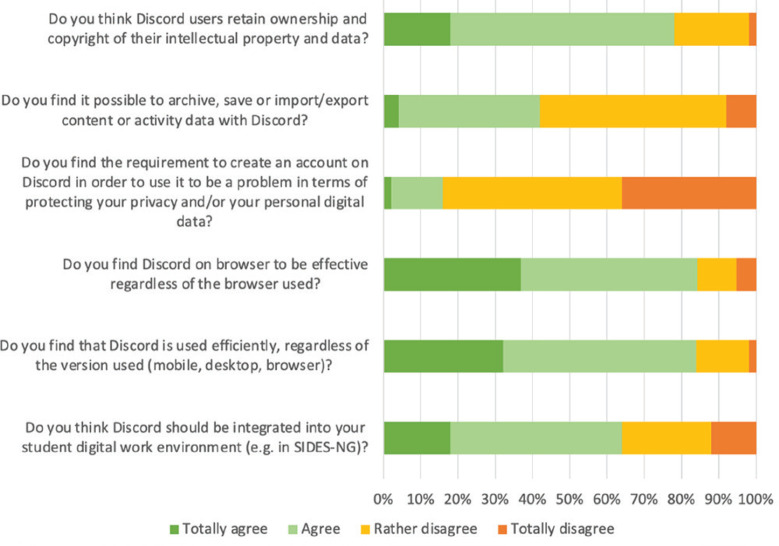
Students’ perception of Discord’s technological qualities and management of personal data

Discord seems to be effective regardless of the version used, and more than 60% of respondents believed that it should be integrated into their learning environment. In general, respondents did not find Discord’s management of personal data to be a problem. Their opinions regarding the possibility for the user to manage their data were very mixed (Figure 3).

### Community of inquiry

**Figure 4:**
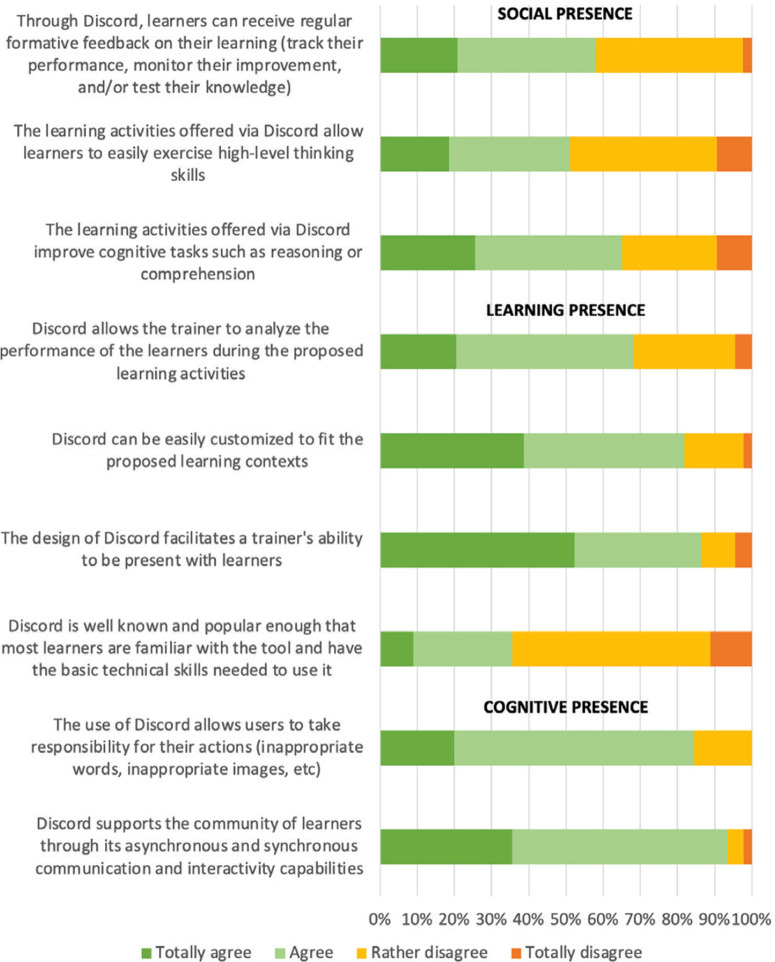
Analysis of the social, learning and cognitive presences enabled by the use of Discord

The three dimensions of analysis, which are social presence, learning presence and cognitive presence are represented in Figure 4. While participating students did not think Discord was popular enough to generate no problems with its use, over 80% found that the tool empowers users and encourages collaboration. More than 80% believed that Discord can be adapted to different learning contexts, and facilitates the connection between instructor and learners. However, opinions were less clearcut about the facilitation of learning performance analysis. For half of the respondents, the tool did not especially offer the possibility of exercising high-level thinking skills. Overall, it did not seem to be an obvious facilitator of cognitive presence.

### Projection

**Figure 5:**
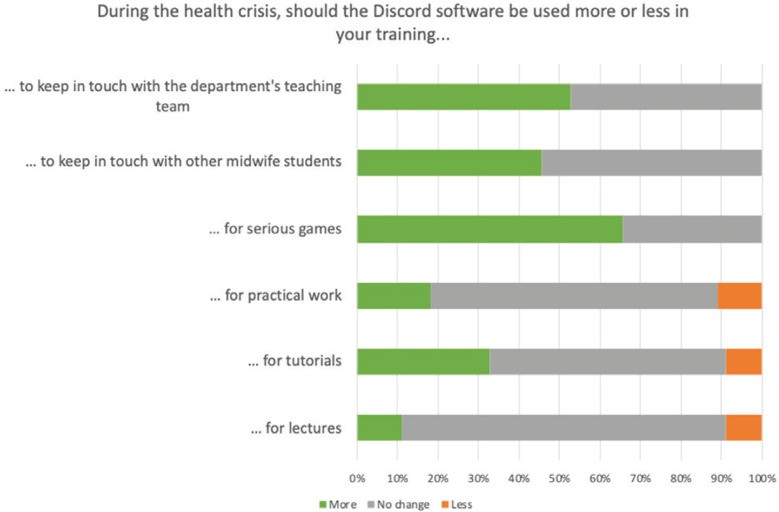
Change of use of Discord during the health crisis

Finally, even though an entire class of students did not use Discord during this year of distance learning, and even though there was fear of having too many different sources of information with the use of Discord on a more regular basis, they felt that the use of this tool could have been at least the same across contexts (Figure 5).

## DISCUSSION

### A consistent declarative study

Our work shows unexpected results, but the internal validity of the study does not seem to be compromised, and despite some biases, the results remained consistent.

The participation rate of 55.6% is explained primarily by the fact that one class was not asked to use Discord at all by their referent teacher. These 37 students probably did not feel involved in the study despite the fact that their opinion about Discord was well sought, regardless of user experience of the target population. If we relate the 86 responses to the 114 students who actually used the Discord Department’s server during the lockdown period, then the participation rate rises to 75%.

There is a lack of consistency between the fact that respondents said they were not familiar enough with Discord to need help to use it and the fact that it is easy to use. This is probably due to a particular inductive sentence pointing more to the popularity of the tool than to its ease of use. Confirmation bias may also have influenced these responses: Discord has a strong image as a tool for gamers. This is consistent with the interest that students find in this tool for making serious games.

It is interesting to note what kind of serious games have been used with Discord and are usually offered to midwifery students in Grenoble-Alpes University. These are not video games, as classically described, but the ‘gamification’ of educational activities. The idea is to bring a playful dimension to certain learning activities in order to engage students in active learning. For example, for definitional learning, they are offered ‘Time's Up’ types of games; while for procedural learning, they are offered ‘Parsely Games’ types. These are always group games, adapted from existing game mechanics and offering a serious part adapted to a precise moment of their learning.

Data management when using Discord did not seem to be a concern for the participating students. It should be noted, however, that these students did not have a minimum education in computer and internet skills. Thus, they were probably not very competent to judge correctly whether Discord is a tool that respects users’ personal data.

Finally, it should be noted that these students come from a first year of health studies in which all lectures are dematerialised^8^: they are therefore well equipped with computers. Moreover, the faculty offers material and logistical support in case of problems with their hardware or software. Thus, it is logical that they agreed with a more integrated deployment of Discord, and agreed to increase its use for certain distance activities such as serious games.

### Results consistent with the literature

The objective of this evaluation was to determine whether Discord met the standards of software dedicated to learning, and to evaluate the satisfaction of students with its use. Our main results show that, according to respondents’ perceptions, Discord is functional, accessible, well designed, and effective whatever the version. Participating students did not think that personal data management was a problem with this tool; and they considered that social presence and learning presence can be improved by its use. Finally, the only unattractive point remains the cognitive presence, since students did not find Discord especially helpful regarding high-level learning. However, serious games, which are popular with students on Discord, are recognized as a means of deeper learning^9^.

These students therefore thought that Discord was particularly useful in order to keep in touch with other students, but also with the department’s teaching staff, in the context of distance learning. They did not seem to find the tool interesting for following lectures, but agreed to increase its use in the context of serious games.

In this context, Discord was described by the respondents as a social network, allowing students to connect with each other. The literature has shown that the use of social networks activates the reward system^10^, which is also the case for the gamification of learning^11^. The fact that students reported that they found Discord interesting for making serious games seems to be related to their perception of this tool.

Our experience in using Discord during the pandemic is not an isolated one. Some pedagogical teams have shown the value of using Discord as a communication environment between students and teachers^12^ or to improve learning processes^13,14^. Some experiments have even broken new ground by showing that Discord can change students’ attitudes during lessons: they are more active, interactive, motivated and creative when the Discord application is activated^15,16^. However, user-centered evaluation of Discord as a learning tool has never been done before.

### Discord: A useful tool during midwifery studies

Midwifery studies in France last 5 years. The first year is a very theoretical and selective year, as only 15% of students are admitted to continue into the second year. The following two years are focused on learning physiology in midwifery, with a direct link between the courses and the validation of internships. There is thus an important learning of clinical reasoning from the beginning of the studies. The last two years are focused on learning how to detect pathologies, and a much larger number of internships. Understanding the links between clinical pictures and theory through deep learning is therefore an important part of the cognitive processes of midwifery students.

The creation of serious games can help anchor this learning. In the context of a health crisis, it is logical that confined students would want to be able to stay in contact with each other and with the teaching staff. Similarly, in the stressful context of a global pandemic, it is logical that they are looking for ways to relax even during their studies, and therefore they are looking for serious games.

In a more global context, the contributions of flipped classrooms, hybrid learning and gamification in health studies are undeniably useful (increase in the grade, motivation level, satisfaction, engagement, etc.)^17-20^. The results of our study show that it is possible to use a communication tool as simple and ergonomic as Discord in order to set up various learning activities. This conclusion is all the more true if we consider other health crises to come, involving periods of confinement for students^21^.

### Strengths and limitations

This declarative study had very good participation and is representative of user opinions in the local context of Grenoble-Alpes University and the COVID-19 pandemic, even though it was a small-scale study. While the popularity of the tool may have influenced some of the responses, this did not prevent it from being shown to be suitable for use in the education of midwifery students, at least for some aspects of the education.

There are few biases or confounding factors in this work. The respondents were students who were generally comfortable with the computer tool and who had an opinion of Discord that is fairly consistent with general opinion.

## CONCLUSIONS

The use of Discord in the training of health professionals should be considered when the training has to be done at a distance. Our study showed that it follows, according to the users, the standards of software dedicated to learning and in the literature there is interest in terms of being a communicative and motivating tool. Moreover, this tool is very popular with students, can give them the impression of better results and above all that the institution reacts more quickly to their requests^22^.

While distance learning has its limitations (distractibility, access to technology, interaction, etc.)^23^, in the context of a population with easy access to technology, Discord makes it possible to compensate for the lack of interaction that is exacerbated by confinement during the pandemic.

Since September 2021, the Discord software offers a new section entirely dedicated to training institute servers. This is the measure of the impact of the pandemic on the means and ways of training students: when supply adapts to demand, the market has proved its worth^24^.

## Data Availability

The data supporting this research are available from the author on reasonable request.
